# Ensheathing glia promote increased lifespan and healthy brain aging

**DOI:** 10.1111/acel.13803

**Published:** 2023-02-24

**Authors:** Lihong Sheng, Emily J. Shields, Janko Gospocic, Masato Sorida, Linyang Ju, China N. Byrns, Faith Carranza, Shelley L. Berger, Nancy Bonini, Roberto Bonasio

**Affiliations:** ^1^ State Key Laboratory of Medical Neurobiology and MOE Frontiers Center for Brain Science Institutes of Brain Science, Fudan University Shanghai China; ^2^ Epigenetics Institute University of Pennsylvania Perelman School of Medicine Philadelphia Pennsylvania USA; ^3^ Department of Cell and Developmental Biology University of Pennsylvania Perelman School of Medicine Philadelphia Pennsylvania USA; ^4^ Department of Urology and Institute of Neuropathology Medical Center–University of Freiburg Freiburg Germany; ^5^ Medical Scientist Training Program University of Pennsylvania Perelman School of Medicine Philadelphia Pennsylvania USA; ^6^ Neuroscience Graduate Group University of Pennsylvania Philadelphia Pennsylvania USA; ^7^ Department of Biology University of Pennsylvania Philadelphia Pennsylvania USA; ^8^ Department of Genetics University of Pennsylvania Perelman School of Medicine Philadelphia Pennsylvania USA

**Keywords:** aging, brain, drosophila, glia

## Abstract

Glia have an emergent role in brain aging and disease. In the *Drosophila melanogaster* brain, ensheathing glia function as phagocytic cells and respond to acute neuronal damage, analogous to mammalian microglia. We previously reported changes in glia composition over the life of ants and fruit flies, including a decline in the relative proportion of ensheathing glia with time. How these changes influence brain health and life expectancy is unknown. Here, we show that ensheathing glia but not astrocytes decrease in number during *Drosophila melanogaster* brain aging. The remaining ensheathing glia display dysregulated expression of genes involved in lipid metabolism and apoptosis, which may lead to lipid droplet accumulation, cellular dysfunction, and death. Inhibition of apoptosis rescued the decline of ensheathing glia with age, improved the neuromotor performance of aged flies, and extended lifespan. Furthermore, an expanded ensheathing glia population prevented amyloid‐beta accumulation in a fly model of Alzheimer's disease and delayed the premature death of the diseased animals. These findings suggest that ensheathing glia play a vital role in regulating brain health and animal longevity.

## Graphical abstract

Ensheathing glia decrease in number during *Drosophila melanogaster* aging, with dysregulated expression of genes involved in lipid metabolism and apoptosis. Inhibition of apoptosis in ensheathing glia extended lifespan, improved climbing ability of aged flies, and prevented amyloid‐beta accumulation in a fly model of Alzheimer's disease.

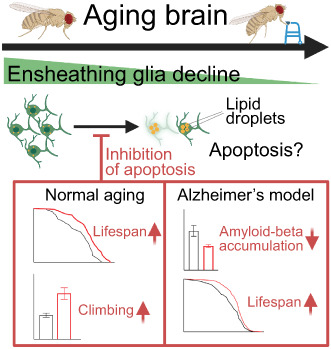

## 1 INTRODUCTION

During aging, the brain undergoes broad structural and physiological alterations that result in a progressive loss of functional abilities (Burke & Barnes, 2006; Mattson & Arumugam, 2018). Aging‐related research in the brain has largely been focused on the alterations in neurons. However, recent studies have shown that glial cells exhibit a great degree of age‐related plasticity, both at the level of gene expression and cell type composition (Soreq et al., 2017; Ximerakis et al., 2019). Glia are vital to the nervous system, as they regulate brain homeostasis in both health and disease. The main function of glia is to provide structural and metabolic support for neurons, activate immune responses in case of infection, and regulate synaptic communication and plasticity (Allen & Lyons, 2018; Freeman, 2015; Zuchero & Barres, 2015). Dysfunction of glia is associated with aging (Conde & Streit, 2006; Matias et al., 2019; Salas et al., 2020; Tremblay et al., 2012) and with brain disease, including neurodevelopmental disorders such as autism (Estes & McAllister, 2015; Petrelli et al., 2016), as well as neurodegenerative disorders such as Alzheimer's disease (Dzamba et al., 2016; Holtzman & Ulrich, 2019; Hong et al., 2016). With our growing understanding of how glia function in the brain, targeting glial cells may be a strategy to promote healthy brain aging and treat age‐related neurological disease (Minhas et al., 2021; Pluvinage et al., 2019).

From *Caenorhabditis elegans* to *Drosophila melanogaster* to mammals, the number and diversity of glial cells increase with brain complexity, but the basic morphology and functions of glia are broadly conserved across the animal kingdom (Freeman, 2015; Hartline, 2011; Nave & Werner, 2021; Raiders et al., 2021). One such function might be to combat harmful age‐associated processes. In *C. elegans*, four astrocyte‐like sheath glia (CEPsh) regulate longevity via neuropeptide signaling (Frakes et al., 2020). Glial suppression of NF‐κB immune signaling in *Drosophila* extends lifespan (Kounatidis et al., 2017). A previous study from our group discovered an age‐associated decline of neuroprotective ensheathing glia in the model ant *Harpegnathos saltator* (Sheng et al., 2020). We also discovered that the conversion of short‐lived *Harpegnathos* workers to long‐lived pseudo‐queens resulted in the expansion of the ensheathing glia population and a slower decline of its numbers during aging, suggesting the possibility that ensheathing glia dynamics can contribute to lifespan regulation. Ensheathing glia play important roles in debris clearance during development and injury, similar to the function of microglia in mammals (Doherty et al., 2009), although they have a very different developmental origin. However, the question of whether ensheathing glia numbers directly affect longevity has not been explored.

Here, we used *Drosophila* genetics coupled with imaging, high‐throughput sequencing, and functional assays to demonstrate that reverting the age‐associated loss of ensheathing glia promotes healthy brain aging and extends lifespan in a variety of settings, including in an Alzheimer's disease model.

## 2 RESULTS

### 2.1 Ensheathing glia, but not astrocytes, decline in number with age in *Drosophila*

**FIGURE 1:**
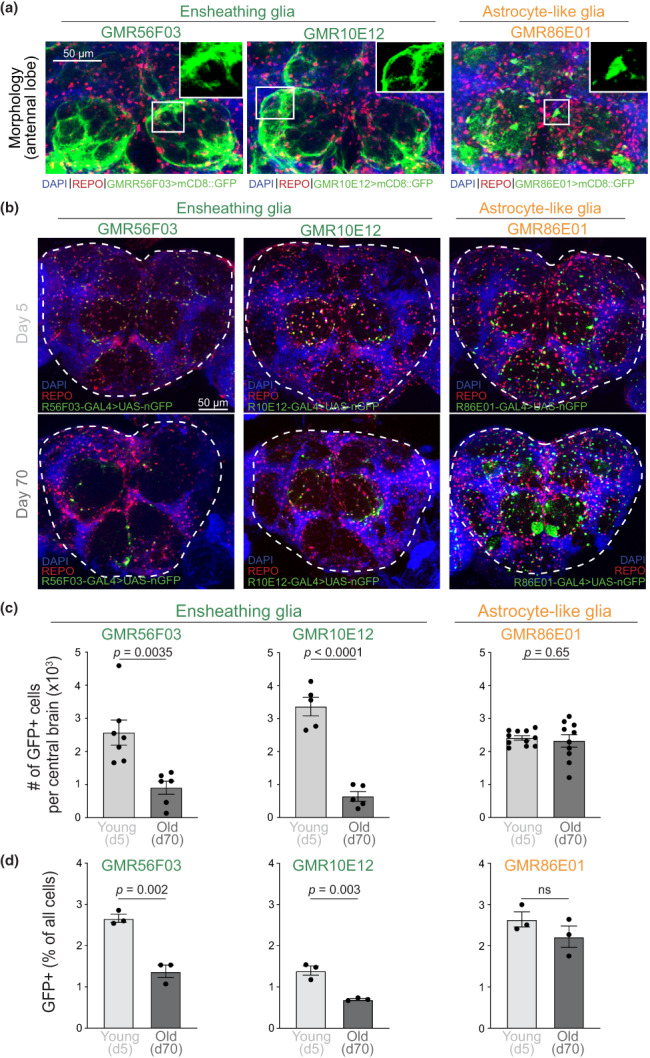
Ensheathing glia cell numbers decline over time. (a) Morphology of glia cells expressing the different genetic drivers in the *Drosophila* antennal lobe. Images are maximum intensity projections from confocal *Z‐*stacks of whole brains from flies expressing membrane‐bound GFP (mCD8::GFP) under the control of *GMR56F03* (left), *GMR10E12* (middle), or *GMR86E01* (right) GAL4 drivers. Brains were counterstained for DAPI (all nuclei) and with a REPO antibody (all glia cells). Zoomed‐in inset shows morphology of the relevant cell types. (b) Brains of 5‐day‐old (top) and 70‐day‐old flies (bottom) with the indicated GAL4 lines driving the expression of nuclear GFP (nGFP) and counterstained for DAPI and REPO. (c) Total number of GFP+ per central brain, as delineated by dashed lines in (b). Individual dots represent independent biological replicates (different brains). Bars represent mean ± SEM. *p* values are from Student's *t* tests. (d) Percentage of GFP+ over total brain cells, as determined by flow cytometry. Individual dots represent independent biological replicates (different brains). Bars represent mean ± SEM. *p* values are from Student's *t* tests.

To study glia population dynamics during aging, we utilized established *Drosophila* GAL4‐driver lines; two with predominant expression in ensheathing glia [*GMR56F03‐GAL4* (Kozlov et al., 2020; Kremer et al., 2017; Peco et al., 2016; Stahl et al., 2018; Wu, Li, et al., 2017) and *GMR10E12‐GAL4* (Wu, Li, et al., 2017)] and one specific for astrocyte‐like glia [*GMR86E01‐GAL4* (Kremer et al., 2017; Saikumar et al., 2020; Sengupta et al., 2019)]. The two ensheathing glia drivers showed comparable expression patterns in the antennal lobe [as determined with a *UAS‐mCD8::GFP* reporter (Wu, Li, et al., 2017)], resembling the known distribution of ensheathing glia cells in this brain region (Doherty et al., 2009; Kremer et al., 2017), whereas the astrocyte‐specific driver was active in a distinct REPO+ cell population of star‐shaped cells (Figure 1a). We next expressed nuclear GFP (nGFP) under the control of the ensheathing glia or astrocyte‐like glia drivers (Figure 1b) and quantified GFP+ cells within the central brain (Figure 1b, dashed lines) by immunofluorescence. Consistent with our previous findings (Sheng et al., 2020), the number of ensheathing glia in the central brain decreased in old (day 70) compared to young (day 5) flies, regardless of the driver used and both as a percentage of total glia cells (REPO+) or as absolute numbers (Figure 1c and Figure S1a, left and middle). The proportion of astrocyte‐like glia remained approximately the same (Figure 1c and Figure S1a, right), and the total number of REPO+ glia cells did not change significantly (Figure S1b), indicating that the observed age‐associated decline in ensheathing glia was not associated with depletion of all glial cell types.

A recent study reported a decrease in the total number of ensheathing glia cell bodies and the extension of their cytoplasmic processes in the antennal lobe of old (51–54 days) compared to young (4–7 days) flies (Cai et al., 2021). We analyzed multiple brain regions and found that, in addition to the antennal lobe, the decline of ensheathing glia also affected the subesophageal zone, as well as the remaining portion of the central brain (Figure S1c,d), indicating that this phenomenon is not localized to a single brain structure.

We sought to confirm these findings with an orthogonal approach independent of imaging and immunofluorescence. We obtained single‐cell suspensions from young and old brains of the different reporter lines and analyzed them by flow cytometry. The percentage of total brain cells expressing GFP in young flies ranged from 1 to 3%, depending on the GAL4 driver line (Figure S1e), consistent with the approximate estimates for these cell types obtained by single‐cell RNA‐seq (Croset et al., 2018; Davie et al., 2018) and previous imaging studies (Kremer et al., 2017). A substantial decline in ensheathing glia frequency in old vs. young brains was also evident when using this imaging‐independent approach (Figure 1d, left and middle), whereas the frequency of astrocyte‐like glia did not change (Figure 1d, right).

It is theoretically possible that repression of the GAL4 drivers in old age caused the reduction in GFP signal, rather than a decrease in the number of ensheathing glia cells. This seemed unlikely, given that the decreased in numbers was quantified using two independent ensheathing glia drivers and that GFP intensity per cell was higher in ensheathing glia from old brains as compared to young brains (Figure S2a). Nonetheless, to formally exclude driver downregulation as a potential confounder, we employed the G‐TRACE genetic labeling strategy (Evans et al., 2009). In this system, RFP is expressed in response to the real‐time activity of the GAL4 driver, whereas GFP is permanently activated by GAL4 expression via DNA recombination and continues to be expressed even if the GAL4 driver becomes silenced (Figure S2b).

Using the *GMR56F03* ensheathing glia driver, we found that the RFP+ cells, which express the reporter under direct control of the GMR56F03‐GAL4 driver, decreased in old flies (Figure S2c,d top), consistent with our previous results using GFP as reporter (Figure 1). If ensheathing glia cells dowregulated the *GMR56F03* driver in older brains, they would appear as GFP+ cells that express lower or no RFP (RFP^lo/−^). We did not observe an increased number of these cells in old flies (Figure S2c,d, bottom), excluding the possibility that driver downregulation over time is responsible for our observations.

Overall, our results show that the numbers of ensheathing glia but not astrocyte‐like glia cells decline as flies age, both as relative fraction of total REPO+ glia cells and as absolute numbers per brain, as determined with two orthogonal methods: imaging and flow cytometry.

### 2.2 Old ensheathing glia upregulate genes for lipid metabolism and apoptosis

**FIGURE 2:**
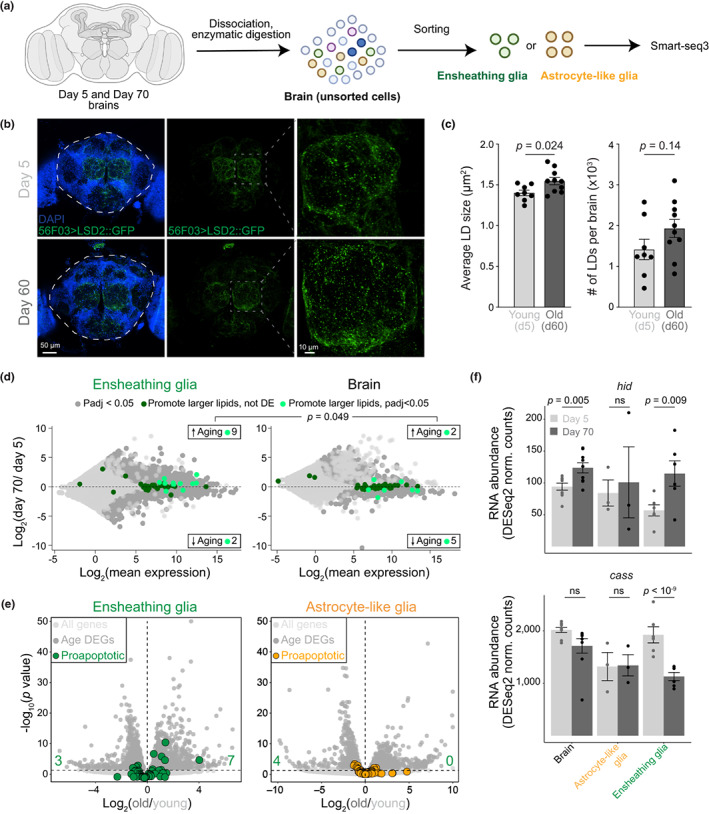
Changes in lipid metabolism and apoptosis in old ensheathing glia. (a) Experimental scheme. Created with BioRender.com. (b) Projections of confocal *Z*‐stacks from whole mount brains of 5‐day‐old (top) and 60‐day‐old flies (bottom) expressing the LD marker, LSD2::GFP from the ensheathing glia driver GMR56F03. DAPI staining was used to visualize nuclei. The dashed white lines indicate the central brain region used for quantification in (c). Panels to the right show a zoomed‐in image of the region in the dashed gray box. (c) Quantification of size (left) and number (right) of 56F03 > LSD2::GFP foci in the central brains of young (Day 5) and old (Day 60) flies. Points represent individual brains. Bars show mean ± SEM. *p* values are from Student's *t* tests. (d) MA plots showing transcriptional differences between ensheathing glia (left) or whole brains (right) from 5‐ and 70‐day‐old flies, combining data from both driver lines (56F03 and 86E01). Genes that promote accumulation of large LDs (Guo et al., 2008) are highlighted in dark green; genes differentially expressed in each tissue (adjusted *p* < 0.05) are in light green. The enrichment of upregulated lipid promoting genes in old ensheathing glia (9/11) compared to old brain (2/7) was significant (*p* = 0.049) according to a Fisher's exact test. (e) Volcano plots showing differential gene expression in young and old ensheathing glia (left) or astrocytes (right). Differentially expressed genes (adjusted *p* < 0.05) are in dark gray; all others are in light gray. Genes annotated as “positive regulators of apoptosis” are highlighted in color. (f) Expression (normalized counts) of *hid* and *cass* in the whole brain, sorted astrocyte‐like glia, and sorted ensheathing glia in young and old flies. Bars show the mean ± SEM. *p* values are from Student's *t* tests.

To identify molecular changes that might underpin the decline of ensheathing glia with age, we sorted GFP+ cells from young (Day 5) and old (Day 70) brains using the GAL4 driver lines above (see Figure 1a) and obtained their genome‐wide gene expression profiles using a modified Smart‐seq3 protocol (Hagemann‐Jensen et al., 2020) (Figure 2a). As controls, we also profiled astrocytes, as well as unsorted single‐cell suspensions from the same brains. The reproducibility between biological replicates was high (Figure S3a), and principal component analysis (PCA) showed a clear separation of expression between the ages along the second dimension, whereas the first PC discriminated, as expected, between whole brains and sorted glia (Figure S3b).

We confirmed that the sorted glia cells had low expression of a neuronal marker (*elav*) and high expression of the pan‐glia marker *repo* (Figure S3c). Furthermore, the sorted glia subtypes expressed markers consistent with their identity: cells sorted using the astrocyte driver GMR86E01‐GAL4 expressed high levels of *alrm* (Davie et al., 2018; Doherty et al., 2009) (Figure S3c), whereas cells sorted using the GMR10E12‐GAL4 or GMR56F03‐GAL4 ensheathing glia drivers expressed comparatively higher levels of ensheathing glia markers *egr* and *sgl* (Figure S3c) (Davie et al., 2018; Sheng et al., 2020). We also detected *alrm* in sorted ensheathing glia as well as *egr* and *sgl* in sorted astrocytes although at lower levels compared to their expression in the appropriate subtype (Figure S3c). This was consistent with published single‐cell RNA‐seq analyses (Davie et al., 2018). When considering all markers for the different glia subtypes identified by single‐cell RNA‐seq, PCA clearly distinguished between the two ensheathing glia drivers on one hand and the astrocyte‐like driver, on the other (Figure S3d), supporting the specificity of the GAL4 drivers.

Next, we compared gene expression profiles from the different glia types sorted from old or young flies. Gene ontology analyses of genes differentially expressed in young vs. brains revealed enrichment for terms related to metabolism and mitochondrial function (Table S1) consistent with their important roles in the aging process (Sun et al., 2016). GO terms enriched in genes regulated in young vs. old glia subtypes included similar terms related to metabolism (“*cellular respiration*”, adjusted *p* = 2.6 × 10^−5^) as well as terms related to neuronal and glial function and turnover (“*synaptic signaling*,” adjusted *p* = 2 × 10^−13^; “*gliogenesis*,” adjusted *p* = 2.3 × 10^−4^). Interestingly, they also included “*lipid homeostasis*” (adjusted *p =* 0.01) and “*lipid storage*” (adjusted *p* = 0.03) The gene most strongly downregulated with age in GMR56F03‐expressing ensheathing glia was *Nplp2* (Figure S3e, top; Table S2), which encodes an exchangeable apolipoprotein that controls lipid flow in the gut (Rommelaere et al., 2019). *Nplp2* deficiency leads to the accumulation of lipids in cells (Rommelaere et al., 2019). Conversely, aged ensheathing glia exhibited increased expression of *Fatp1* (Figure S3e, bottom; Table S2), a fatty acid transporter that regulates lipid droplet (LD) accumulation in the opposite direction (Liu et al., 2017). These results suggest that old ensheathing glia might accumulate lipids, a phenomenon involved in neurodegeneration in *Drosophila* (Liu et al., 2015).

A lipid stain, BODIPY, revealed the presence of LDs co‐localized with mCD8::mCherry expressed in ensheathing glia with the GMR56F03 driver (Figure S4a). To confirm the presence of LDs in ensheathing glia and measure their dynamics during aging, we expressed a genetic marker of LDs, LSD2::GFP (Gronke et al., 2003; Kis et al., 2015; Welte et al., 2005), specifically in ensheathing glia (Figure 2b). By quantifying images from young and old brains, we determined that LDs marked by LSD2::GFP foci were significantly larger (*p* = 0.024) in ensheathing glia cells from old brains (Figure 2c, left) and also showed a trend toward increased total numbers (Figure 2c, right). Given that old brains contain a much smaller number of ensheathing glia cells, our quantifications suggest the accumulation of LDs in these cells over time.

We compared our RNA‐seq data from sorted ensheathing glia with published data of genomic screen genes involved in LD formation (Guo et al., 2008). In total, 11 genes that promote the accumulation of large LDs were differentially expressed in ensheathing glia, with 9 (82%) upregulated in old cells (Figure 2d, left, and Table S3). Interestingly, a similar skew was not observed in the old brain as a whole (Figure 2d, right, and Table S4), suggesting that lipid metabolism is affected specifically in old ensheathing glia (*p =* 0.049, Fisher's exact test).

In the aging mouse brain, phagocytic microglia accumulate LDs and acquire a dysfunctional state marked by genome‐wide changes in transcription, including dysregulated expression of phagocytosis genes (Marschallinger et al., 2020). We cross‐analyzed the transcriptome of LD‐high and LD‐low microglia from 18‐month‐old mouse brains against the gene expression profile of sorted young and old *Drosophila* ensheathing glia cells. Despite the large evolutionary distance between insects and mammals, we detected an overall upregulation of genes associated with the high LD phenotype in old ensheathing glia (Figure S3f), suggesting that age‐related dysregulation of lipid metabolism in glia is conserved across species.

During stress, aging, and neurodegenerative disease glia cells—and less often neurons—accumulate LDs (Bailey et al., 2015; Farmer et al., 2020; Henne et al., 2018; Liu et al., 2015, 2017; Smolic et al., 2021). Furthermore, increased lipid droplet formation following the induction of cell death has been widely observed (Farmer et al., 2020; Hakumaki et al., 1999; Shyu Jr. et al., 2018). The GO terms “*programmed cell death*” and “*apoptotic process*” were both significantly enriched (adjusted *p* = 0.02 and 0.047, respectively) in genes differentially expressed in old vs. young ensheathing glia (Table S1). Further analyses revealed that 7 out of 51 genes annotated with the GO term “*positive regulation of apoptotic process*” (GO:0043065) were upregulated in old ensheathing glia (Figure 2e, left), whereas none of them were upregulated in old astrocytes (Figure 2e, right). For example, old ensheathing glia upregulated the pro‐apoptotic gene *hid* (Grether et al., 1995) (Figure 2f, top) and downregulated in the anti‐apoptotic gene *cass* (Rigou et al., 2009) (Figure 2f, bottom).

Together, our data indicate that ensheathing glia cells from older brains are characterized by a distinct transcriptional and phenotypic state, which include dysregulated lipid metabolism, accumulation of LDs, and activation of a subset of pro‐apoptotic genes.

### 2.3 Inhibiting apoptosis of ensheathing glia extends lifespan

Based on our transcriptomic analyses, we hypothesized that apoptosis might be responsible for the decline of ensheathing glia in old flies. Staining for annexin V, which can be used to detect apoptosis in *Drosophila* brains (van den Eijnde et al., 1998; Venkatachalam et al., 2008), revealed that apoptosis could be readily detected in a fraction of ensheathing glia cells in young brains and that this fraction was significantly (*p* = 0.003) increased in old brains (Figure S4b).

We reasoned that if apoptosis causes loss of ensheathing glia cells in old brains, the expression of an apoptosis inhibitor in these cells might stabilize their numbers during aging. To test this hypothesis, we utilized the baculoviral apoptosis inhibitor *p35*, which inhibits programmed cell death in *Drosophila* triggered by *hid* (Grether et al., 1995), which was one of the apoptotic genes upregulated in ensheathing glia cells from old brains (see Figure 2f). This strategy has been successfully used to prevent programmed cell death in a variety of species (Hay et al., 1994; Sugimoto et al., 1994), and, importantly, blocking apoptosis with p35 still allows normal cellular function (Davidson & Steller, 1998). Because GMR56F03‐GAL4 is a more widely employed line to drive expression in ensheathing glia cells (Kozlov et al., 2020; Kremer et al., 2017; Peco et al., 2016; Stahl et al., 2018; Wu, Li, et al., 2017) and expresses higher levels of ensheathing glia markers compared to GMR10E12 (Figure S5a), without off‐target expression in gut or ovary (Figure S5b), we focused on this ensheathing glia driver for the subsequent functional experiments.

**FIGURE 3:**
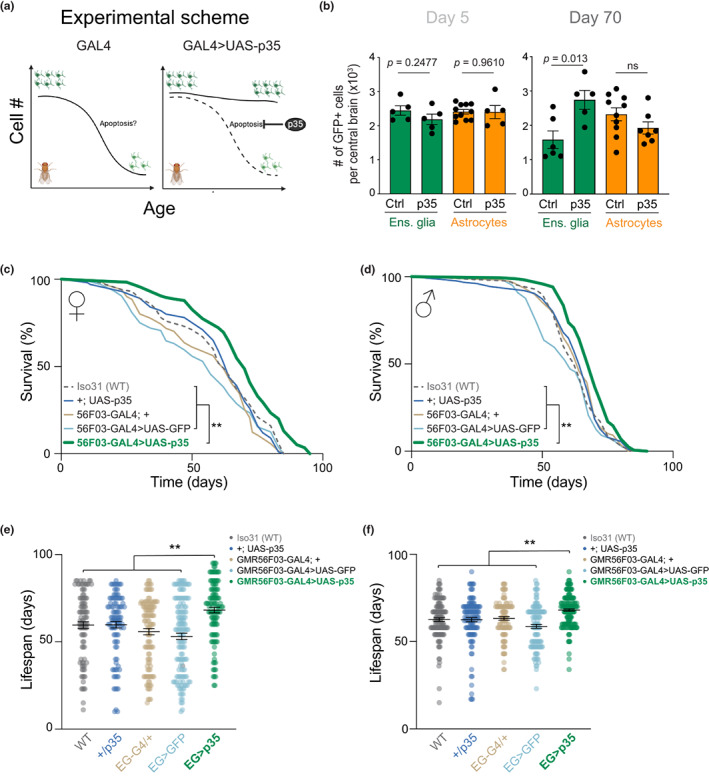
Inhibition of apoptosis in ensheathing glia extends lifespan. (a) Experimental scheme. Drivers for different glia subtypes were used to express the apoptosis inhibitor *p35*. Created with BioRender.com. (b) Quantification of ensheathing glia cells (green bars) or astrocytes (orange bars) in 5‐day‐old (left) and 70‐day‐old (right) brains, with or without expression of p35. Expression of UAS‐GFP and UAS‐p35 was driven by GMR56F03‐GAL4 or GMR86E01‐GAL4 for ensheathing glia or astrocytes, respectively. Quantification was performed as in Figure 1. Dots show individual brains. Bars represent mean ± SEM. *p* values are from Student's *t* tests. (c, d) Lifespan of female (c) or male (d) flies expressing *p35* in ensheathing glia (thick green line) and all relevant controls. The 56F03 > p35 curve was compared against each control with log‐rank Mantel‐Cox tests adjusted for multiple comparisons with the Bonferroni method, all of which gave *p* values <0.01 (**). (e, f) Lifespan distribution of individual females (e) and male (f) flies expressing *p35* in ensheathing glia compared to control flies. The black lines represent the mean ± SEM. The 56F03 > p35 group was compared to each control group with one‐way ANOVA followed by Bonferroni's multiple comparisons test. All comparisons gave *p* values <0.01 (**).

We expressed *p35* in ensheathing glia using the GMR56F03‐GAL4 driver or in astrocyte‐like cells using GMR86E01‐GAL4 and monitored the relative abundance of the targeted cell population (Figure 3a). In young, 5‐day‐old flies, *p35* expression did not significantly affect the numbers of ensheathing glia or astrocytes (Figure 3b and Figure S5c). However, in 70‐day‐old brains, *p35* expression was sufficient to more than double the number of ensheathing glia cells, whereas no change was observed in astrocytes (Figure 3b and Figure S5d). A significant expansion in ensheathing glia numbers, but not of astrocyte numbers, was observed in all individual brain regions analyzed (Figure S5e–g). These results suggest that p35 acts specifically to inhibit the natural apoptosis of ensheathing glia cells in old brains, rather than by causing an indiscriminate increase of cell number.

Our previous work in *Harpegnathos* ants (Sheng et al., 2020) as well as recent studies in other ants and bees (Li, Wang, et al., 2022; Zhang et al., 2022) reported a correlation between ensheathing glia expansion and increased life expectancy, but no causal link could be established. Having arrested the decline in ensheathing glia numbers by expressing *p35*, we next measured lifespan to determine whether the increased number of ensheathing glia in old brains would improve longevity. Remarkably, expression of the apoptosis inhibitor p35 in ensheathing glia significantly increased lifespan in both female and male flies compared to all relevant controls (Figure 3c–f), which included Iso31 wild type flies (WT), flies carrying the driver (GMR56F03‐GAL4; +) or *p35* gene in isolation (+; UAS‐p35), as well as GMR56F03 > UAS‐GFP flies expressing nGFP from the same ensheathing glia driver used for *p35* (see Figure S5h for genotypes and numbers). To avoid confounding background effects on lifespan, all lines used for this experiment were backcrossed ≥6 times to an isogenic wild type control (see Methods). As an additional control, expression of p35 in astrocytes using the GMR86E01 driver did not result in extended lifespan (Figure S5i–l).

### 2.4 Increased ensheathing glia numbers promote healthy brain aging

**FIGURE 4:**
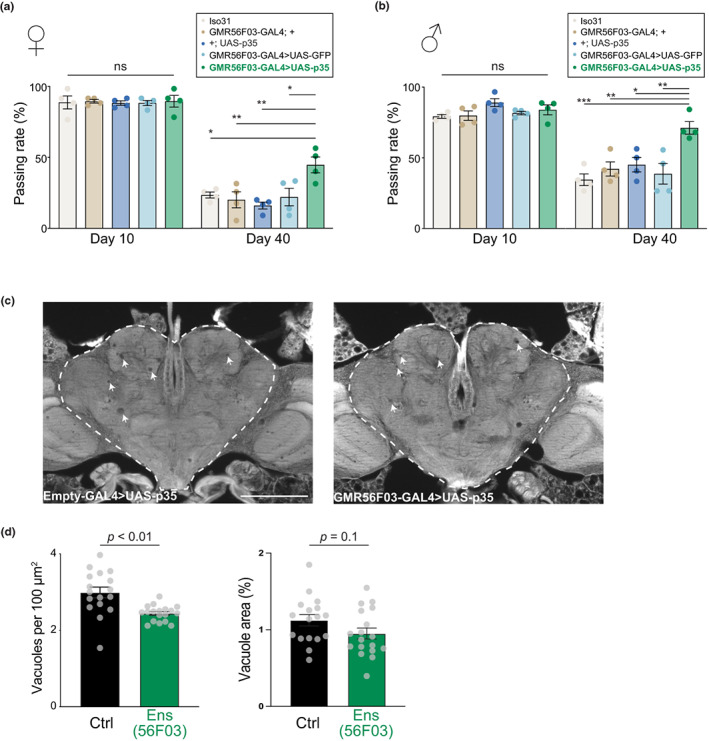
Ensheathing glia promotes functional and morphological healthy brain aging. (a, b) Climbing test for female (a) or male (b) flies at the age of 10 days (left) or 40 days (right). Each circle is a biological replicate (a vial with ≥20 flies). Bars represent mean ± SEM. The 56F03 > p35 group was compared to each control group with one‐way ANOVA followed by Bonferroni's multiple comparisons test. *p < 0.05; **p < 0.01; ***p < 0.001. (c) Histological section from the heads of two representative 70‐day‐old flies, with (right) or without (right) expression of *p35* in ensheathing glia. The arrowheads point to vacuoles. Empty‐GAL4 is a control GAL4 driver with no regulatory region (Bloomington #68384). Scale bar represents 100 μm. (d) Quantification of vacuoles in the central brain, shown by a dashed line in (d). Right, number of vacuoles; left, total vacuole area. Data are from two independent experiments. Points represent separate biological replicates (individual heads). Bars represent mean ± SEM. *p* values are from Student's *t* tests.

We next considered whether the lengthened lifespan of flies with an expanded ensheathing glia population were accompanied by a healthier brain state in old age. First, we determined neuromotor performance in the form of climbing activity (Gargano et al., 2005) at different timepoints. Consistent with the positive effects observed on lifespan, inhibition of ensheathing glia apoptosis significantly improved climbing performance of middle‐aged (40‐day‐old) flies when compared to control flies (Figure 4a,b).

In *Drosophila*, one histological hallmark of aging brains is the appearance of gaps in the tissue, or “vacuoles” (Kang et al., 2017; McGurk et al., 2015). We reasoned that if ensheathing glia contribute to healthier brains with age, this may correspond to a decreased accumulation of vacuoles. Compared to controls, brains from old flies with more ensheathing glia showed a modest, but significant, reduction in the number of vacuoles and a trend towards reduction in the total area occupied by vacuoles (Figure 4c,d). This result cannot be used to discriminate whether ensheathing glia are directly or indirectly responsible for the clearance of vacuoles.

These results demonstrate that preventing the age‐associated loss of ensheathing glia is sufficient to improve neuromotor performance and mitigate the accumulation of histological damage in old brains, suggesting that ensheathing glia contribute to healthy brain aging as determined by both functional and morphological metrics.

### 2.5 Ensheathing glia delay neurodegeneration and death in a model of Alzheimer's disease

**FIGURE 5:**
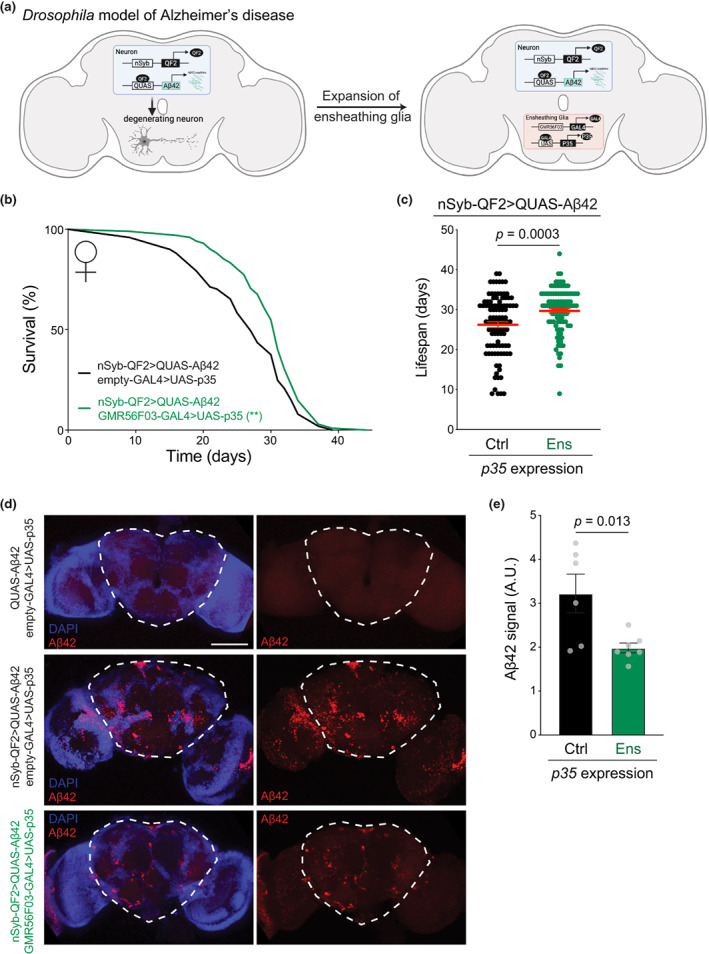
Ensheathing glia expansion prevents Aβ42 accumulation and extends lifespan in a model of Alzheimer's. (a) Experimental strategy to inhibit ensheathing glia apoptosis in a *Drosophila* model of Alzheimer's disease. Created with BioRender.com. (b) Lifespan of female flies expressing Aβ42 in all neurons and with (green, *n =* 102) or without (black, *n* = 101) *p35* expression in ensheathing glia. *p* value is from a log‐rank (Mantel‐Cox) test. **p < 0.01. (c) Lifespan distribution of individual female flies expressing Aβ42 in all neurons and with *p35* in ensheathing glia (“ens”, green) or non‐expressing controls (“ctrl”, black). Genotypes are the same as in (b). Red bars represent the mean ± SEM. *p* value is from a Student's *t* test. (d) Projections of confocal Z‐stacks from whole mount brains of 10‐day‐old females stained with anti‐Aβ42 antibody (red) and DAPI (blue). Top, negative control (QUAS‐Aβ42); middle, flies expressing Aβ42 in all neurons; bottom, flies expressing Aβ42 in all neurons and p35 in ensheathing glia. The scale bar represents 100 μm. (e) Quantification of Aβ42 signal in the central brain (dashed line) from immunofluorescence images as shown in (d). Circles represent individual replicates. Bars represent the mean ± SEM. *p* value is from a Student's *t* test.

Finally, we sought to determine whether increased ensheathing glia numbers would also be sufficient to arrest or delay more aggressive pathological forms of age‐associated neurodegeneration. We utilized a genetic model that recapitulates key features of Alzheimer's disease by expressing the human amyloid‐β 1–42 fragment (Aβ42) in fly neurons using the driver nSyb‐QF2 (Figure 5a). Expression of Aβ42 in *Drosophila* results in age‐dependent molecular and behavioral effects, including Aβ42 accumulation and neuronal hyperexcitability, and ultimately a severely reduced lifespan (Finelli et al., 2004; Ping et al., 2015). Remarkably, expression of the apoptosis inhibitor *p35* in ensheathing glia greatly improved survival (Figure 5b) also in the context of this disease model, with a 15% increase in average lifespan (Figure 5c). Flies expressing *p35* in ensheathing glia displayed a significant reduction of Aβ42 accumulation in the brain (Figure 5d,e) These findings suggest that ensheathing glia reduce Aβ42‐associated lifespan decline by mitigating the accumulation of Aβ peptide, possibly through active removal of Aβ42 via phagocytosis. Consistent with this possibility, antibody stainings revealed the presence of Aβ42 within the cytoplasm of ensheathing glia cells (Figure S6).

Together, our results indicate that ensheathing glia contribute to longevity both in physiological settings by preventing age‐associated structural deterioration of the brain and in pathological settings by mitigating accumulation of toxic peptides and delaying overt neurodegenerative disease.

## 3 DISCUSSION

Glia are an indispensable component of the brain's repertoire of cells and play a key role in maintaining brain health and homeostasis. Many studies in different species have reported changes in glia composition and function with age (Davie et al., 2018; Sheng et al., 2020; Soreq et al., 2017; Ximerakis et al., 2019), but the overall physiological implications of these cellular dynamics remain poorly understood. Here, we report that the number of neuroprotective ensheathing glia declines with age in the *Drosophila* brain and that inhibition of this loss extends animal lifespan likely through improved structural as well as functional integrity of old brains. Furthermore, we show that these neuroprotective functions of ensheathing glia extend beyond normally aging brains and are beneficial in the context of pathological neurodegeneration.

### 3.1 Glia dynamics in aging brains

Aging causes cellular dysfunction and progressive loss of cell numbers. Recent studies indicate that glia are affected early and extensively by the aging process (Salas et al., 2020). In mammalian systems, unbiased and high‐throughput methods have revealed great heterogeneity in microglia, which, however, decreases over the course of development and aging (Li et al., 2019; Masuda et al., 2019, 2020). Besides the limited heterogeneity, microglia appear to switch their function from neuroprotective in the young brain to neurotoxic in the aged brain, where they accumulate LDs (Luo et al., 2010; Marschallinger et al., 2020).

Similar to microglia, the neuroprotective ensheathing glia in *Drosophila* show a declining ability to clear neuronal debris with age (Purice et al., 2017) and accumulate lipid droplets, potentially promoting neurodegeneration (Liu et al., 2015). *Drosophila* glia exhibit an abnormal activation of transcription factors in age, which in turn promotes age‐onset tau pathology (Byrns et al., 2021). Our results show that the number of ensheathing glia decreases in old brains. This conclusion is supported by two independent genetic drivers and two orthogonal quantification methods (imaging, flow cytometry) and it is true both when expressing the numbers in absolute or relative form (Figure 1 and Figure S1). Consistent with our observations, a recent study reported a loss of ensheathing glia in aging brains specifically in the antennal lobe (Cai et al., 2021).

Ensheathing glia from old brains also display an altered transcriptional profile (Figure 2). One possibility is that age causes a deterioration of the cellular functions of ensheathing glia (Hu et al., 2021; Shahidehpour et al., 2021), leading to stasis of intracellular pathways, lipid accumulation, followed by programmed cell death. The notion that apoptosis is responsible for the age‐associated decline of ensheathing glia is supported by genomic data, flow cytometry, and functional evidence: (1) pro‐apoptotic genes, including *hid* are significantly upregulated in old ensheathing glia but not in old astrocytes (Figure 2e), (2) the fraction of ensheathing glia staining for the apoptotic marker annexin V increase in old brains (Figure S4b), and (3) expression of the anti‐apoptotic gene *p35* reverts the loss of ensheathing glia and not astrocytes in old brains but has no effect on either glia subset in young brains (Figure 3b). Nonetheless, we cannot exclude alternative or additional explanations for the decline of ensheathing glia, nor do we claim that only ensheathing glia experience changes in cell numbers during aging. Importantly, our central finding that a higher number of ensheathing glia cells in old brains extends lifespan holds regardless of the precise mechanism of their natural decline.

Although they have different morphologies and transcriptional profiles, astrocytes and ensheathing glia have shared developmental origins and, to some extent, defects in one lineage can be compensated by the other (Kato et al., 2020). However, we see no evidence for a compensatory expansion of the astrocyte compartment in older brains with less ensheathing glia (Figure 1c,d), and our rescue experiments indicate that they cannot fully replace the function of the missing ensheathing glia (Figures 3, 4, 5). We cannot, however, exclude that astrocytes or other glia subtypes that we did not directly measure might undergo complex dynamics during aging, including a combination of apoptosis or expansion, perhaps via apoptosis‐induced proliferation (Fan & Bergmann, 2008). These possibilities do not affect our central conclusion that ensheathing glia numbers contribute to healthy brain aging and lifespan regulation.

### 3.2 Phagocytic glia and healthy brain aging

In addition to playing an essential role in the innate immune response, phagocytosis is required for proper neural circuit development and the maintenance of brain homeostasis (Galloway et al., 2019). Glia phagocytosis contributes to defense against pathogens and clearance of degrading axon debris during normal aging as well as disease‐specific protein aggregates, such as amyloid‐β (Aβ) in Alzheimer's disease (Jung & Chung, 2018). In the mammalian brain, two different glial cells, astrocytes and microglia, have been shown to phagocytose synapses, apoptotic cells, cell debris, and released toxic proteins (Damisah et al., 2020; Konishi et al., 2020; Tremblay et al., 2019).

The function and structure of phagocytic glia are dynamic during brain aging and disease, and they contribute in general to healthy brain function (Cheray & Joseph, 2018; Falcon‐Moya et al., 2020; Fuger et al., 2017; Pirttimaki & Parri, 2013; Shemer et al., 2015; Zhou et al., 2019). For example, microglia play a key role in the onset and progression of Alzheimer's disease and related disorders (Dzamba et al., 2016). A growing body of studies has revealed the association of microglia heterogeneity with Alzheimer's disease at single‐cell resolution (Keren‐Shaul et al., 2017; Masuda et al., 2020; Olah et al., 2020; Provenzano et al., 2021). Other studies have shown that recovery of microglia cell function can contribute to rejuvenating older brains. So far, this has been achieved by the inhibition of myeloid EP2 signaling (Minhas et al., 2021) or by reactivating phagocytosis via blockage of CD22 (Pluvinage et al., 2019). However, to our knowledge the relative abundance of phagocytic glia has not been previously manipulated to control the aging process. Our data demonstrate that the inhibition of apoptosis in ensheathing glia, which function as phagocytic glia in the fly, is sufficient to extend lifespan and to promote healthy brain aging.

While it is tempting to speculate that our conclusions might apply to the functionally analogous microglia in mammals, it is important to note that ensheathing glia and microglia have very different developmental origin, which constitutes a limitation in our cross‐species comparison. Particular genetic manipulations cause the appearance of a different phagocytic cell type in the *Drosophila* brain that might resemble mammalian microglia more closely (Stratoulias & Heino, 2015). It would be of interest to investigate whether the cellular and molecular dynamics displayed by aging ensheathing glia could also be observed in these microglia‐type cells.

### 3.3 Conclusions and outlook

In this study, we provide direct evidence that preventing the loss of ensheathing phagocytic glia in *Drosophila* is sufficient to improve lifespan (Figure 3), maintain healthy brain structure and function in normal aging (Figure 4), and protect against neurodegeneration (Figure 5). The precise molecular mechanism by which they do so remains to be investigated. We have previously shown that changes in the number of ensheathing glia cells occur naturally during aging and social reprogramming in *Harpegnathos* (Sheng et al., 2020), and caste differences in ensheathing glia abundance have been independently confirmed in another ant species as well as honeybees (Li, Wang, et al., 2022; Zhang et al., 2022). Intriguingly, the lifespan of *Harpegnathos* workers is dramatically increased (~fivefold) when they are reprogrammed into reproductive pseudo‐queens (“gamergates”) (Ghaninia et al., 2017). This reprogramming is accompanied by a physiological expansion of ensheathing glia, indicating that the abundance of this cell type in the brain can be controlled by natural pathways. The identification of the molecular signals that control the expansion of ensheathing glia in *Harpegnathos* and the potential conservation with *Drosophila* and beyond might offer an avenue to promote healthy brain aging.

## 4 MATERIALS AND METHODS

### 4.1 Fly stocks and husbandry

Stocks were maintained, and experiments conducted at 25°C and 50% humidity on a 12‐h light/dark cycle using standard Bloomington *Drosophila* medium (Nutri‐Fly). Fly stocks used in this study were empty‐GAL4 (Bloomington #68384); GMR56F03‐GAL4 (Bloomington #39157); GMR10E12‐GAL4 (Bloomington #46517); GMR86E01‐GAL4 (Bloomington #45914); UAS‐GFP (Bloomington #4775); UAS‐mCD8GFP (Bloomington #5137); UAS‐p35 (Bloomington #5072); nSyb‐QF2 (Bloomington #51960); QUAS‐Aβ42 (Bloomington #83347); G‐TRACE (Bloomington #28280); UAS‐mCherry (Bloomington #35787). The UAS‐LSD2::GFP line was a generous gift from M. Welte and was obtained from A. Sehgal's laboratory (Li, Haynes, et al., 2022) with his permission. Iso31 was a gift from A. Sehgal (Erion et al., 2016). Strains used in Figures 3c–f and 4a,b were backcrossed to this Iso31 strain six times to remove potential confounding effects from differences in genetic backgrounds.

### 4.2 Survival analysis

For lifespan test, newly eclosed flies, over a 24‐h period, were transferred into fresh food vials. After 48 h, 20 mated females or males were collected under mild CO_2_ anesthesia and transferred into fresh food vials. Flies were maintained at 25°C under the same conditions described above and transferred to new food vials 2–3 times per week, with mortality recorded during each transfer. Mortality curves were statistically analyzed using log‐rank tests in GraphPad Prism 9 and, when necessary, *p* values were corrected for multiple testing using the Bonferroni method.

### 4.3 Whole mount brain immunofluorescence and quantification of glia populations

Fly brains were dissected in cold phosphate buffered saline (PBS) as described (Tito et al., 2016) and fixed in 4% paraformaldehyde for 45 min at room temperature with rotation. Next, the brains were washed twice in PBS and permeabilized with 0.5% Triton‐X in PBS (PBST) for 2 h, then blocked with 5% goat serum in PBST for >1.5 h at room temperature. Brains were incubated with primary antibodies overnight at 4°C in 5% goat serum in PBST, washed in PBST and incubated with fluorescently conjugated second antibody for 2 h, washed with PBST and stained with DAPI for 10 min. Last, sample were washed with PBS, mounted with mounting medium (Vector Laboratories, H‐1000), and imaged on a Leica SPE laser scanning confocal microscope. Antibodies used in this study were anti‐REPO (1:5, DSHB 8D12), anti‐GFP (1:500, ab13970), anti‐RFP (1:1000, Rockland Cat# 600‐401‐379), anti‐Aβ42 (1:500, Biolegend 6E10 Cat# 803004), anti‐chicken IgG (H + L), Alexa Fluor 488 (1:500, ThermoFisher Scientific), and anti‐mouse IgG (H + L), Alexa Fluor 568 (1:500, ThermoFisher Scientific). Images represent maximum intensity projections of *Z*‐stacks from whole brains.

FIJI, FIJI macro, and ilastik were used for imaging processing and quantification. The region used to define the central brain is indicated by dashed lines in the relevant figures. This region corresponds to the whole brain minus optic lobes, and has also been referred to as “non‐visual brain” or “midbrain” in other studies (Croset et al., 2018; Gospocic et al., 2017; Ito et al., 2014). Total glial cells were quantified based on their positivity for REPO antibody staining, whereas different glia populations were quantified based on their signal for anti‐GFP antibody. In all cases, quantification was performed in all *Z* planes for ≥5 brains per condition.

### 4.4 Lipid droplet staining

After staining with anti‐RFP, brains were rinsed several times with PBST and incubated with fluorescently conjugated secondary antibody and 20 μg/mL BODIPY 493/503 (Thermo Fisher Scientific, Cat#D‐3922) for 4 h at RT. Subsequently, brains were washed with PBS three times and stained with DAPI, then rinsed immediately, mounted in mounting medium (Vector Laboratories, H‐1000), and imaged with a Nikon AX confocal microscope.

### 4.5 Quantification of apoptosis and G‐TRACE cell populations by flow cytometry

Brains from young (5 days old) and old (>50 days old) females were dissected, collected into a tube with cold Schneider's medium (SM) containing 1% BSA and washed twice. Next, we added 200 μL of dissociating solution, which included 2 mg/mL collagenase I (Sigma‐Aldrich, SCR103) and 20 μg/mL DNase I (Worthington, Cat#LS002006) in SM containing 1% BSA. Brains were dissociated at 25°C using a shaker at 500 rpm for 1 h and vigorous pipetting every 20 min. Samples were centrifuged at 4°C, 500 g for 5 min to collect the cells and annexin V staining was performed following the protocol in the Apoptosis Detection kit (Yeasen, #40304ES20) with minor modifications. Briefly, cells were resuspended in 100 μL binding buffer with 1 μL AnnexinV‐Alexa Fluor 647 and incubated for 15 min at room temperature. We then centrifuged the cells at 4°C, 500 g for 5 min and resuspended them in 200 μL PBS, then filtered through 100 μm cell strainers and immediately analyzed on a BD AriaII. For the G‐TRACE experiment, samples were filtered through 100 µm cell strainers after dissociation and immediately analyzed on a BD AriaII.

### 4.6 Climbing assay

Assay was performed as previously described (Madabattula et al., 2015; Sun et al., 2013). Groups of 20 files were transferred into an empty vial and allowed to recover for at least 30 min. The vials were tapped to force the flies to the bottom and then the animals allowed to climb for 10 s to pass a midline marked ~4 cm from the bottom of the vial. The number of flies that traversed the midline of the vial within the allotted time was recorded. The procedure was repeated three times for each vial, and the average percentage of flies that successfully passed the midline was calculated as the “climbing rate” for that vial. Four biological replicates were performed per condition.

### 4.7 Fluorescence‐activated cell sorting

Brains (10–15 per replicate) from young (5 days old) and old (70 days old) females were dissected, collected into a tube with cold Schneider's medium (SM) containing 1% BSA and washed twice. Next, we added 200 μL of dissociating solution, which included 2 mg/mL collagenase I (Sigma‐Aldrich, SCR103) and 20 μg/mL DNase I (Worthington, Cat#LS002006) in SM containing 1% BSA with 45 μM actinomycin D to block new transcription during enzyme digestion (Wu, Pan, et al., 2017). Brains were dissociated at 25°C using a shaker at 500 rpm for 1 h and vigorous pipetting every 20 min. Dissociated tissue was filtered through 100 μm cell strainers and sorted on a BD InfluxB with a 100 μm nozzle at the sorting facility of the University of Pennsylvania. Dead cells were excluded by staining with 4,6‐diamidino‐2‐phenylindole (DAPI). GFP+ cells gates were set according to the fluorescence profile of GFP‐ brain tissue. Desired glia populations (~5000 cells per replicate) were directly sorted into TRIzol for RNA extraction.

### 4.8 Paraffin embedding of fly heads and brain vacuole quantification

Heads were obtained by rapid decapitation under anesthesia then fixed in Bouin's solution (Sigma‐Aldrich, HT10132) for 5 days. Fixation was terminated by submersion in leaching buffer (50 mM Tris pH 8.0_RT_, 150 mM NaCl) for 1 h at RT. Heads were then processed through graded EtOH dehydration at the following times and concentrations: 30 min 70%, 30 min 90%, 30 min 95%, 30 min 100%, 30 min 100%, followed by xylenes (2×, 30 min) and finally fixed in paraffin. Heads were blocked and sectioned into 8 μm thick ribbons. Ribbons were deparaffinized by heating at 65°C for 1 h followed by washes in histoclear I and II (VWR; 101412‐876, 10141‐8822) for 5 min. For assessing brain vacuolization, sections were mounted with Cytoseal XYL (ThermoFisher, 8312‐4). Sections were imaged on a Leica DFC360 FX under 10× objective, 1.6× magnification with fixed exposure settings. Fly brain tissue is auto‐fluorescent due to pigmented eyes; thus, signal was detected using an I3 filter cube.

Brain vacuolization was quantified in FIJI as follows: For each brain, numbers of vacuoles and vacuolar area in the central brain region (dashed line in Figure 4c) were quantified and divided by the total are of the central brain region to obtain the density per 100 μm^2^ or % vacuolar areas. Vacuole area was set by manual thresholding. Image acquisition and analysis was performed blind to sample identity.

### 4.9 RNA isolation, library preparation, and sequencing

The RNA was purified with TRIzol and library preparation followed by the Smart‐seq3 protocol (Hagemann‐Jensen et al., 2020) with minor modification. Briefly, purified RNA in Smart‐seq3 buffer (5% PEG8000, 0.1% Triton X‐100, 0.5 U/μL RNase inhibitor, 0.5 μM Smart‐seq3 oligo‐dT primer, and 0.5 mM dNTP) was incubated at 70°C for 2 min to denature the RNA. Next, reverse transcription mix (25 mM Tris–HCl pH 8.3_RT_, 30 mM NaCl, 1 mM GTP, 2.5 mM MgCl_2_, 8 mM DTT, 0.5 U/μL RNase inhibitor, 2 μM TSO and 2 U/μL of maxima H‐minus reverse transcriptase enzyme) was added to each sample. Reverse transcription and template switching were carried out at 42°C for 90 min followed by 10 cycles of 50°C for 2 min and 42°C for 2 min. The reaction was terminated by incubating at 85°C for 5 min. PCR pre‐amplification was performed by directly adding KAPA HiFi HotStart ReadyMix and 0.5 μM forward primer and 0.1 μM reverse primer to a 50 μL reaction volume. The PCR was performed with the following cycle: 3 min at 98°C for initial denaturation, 5–9 cycles (depending on the starting amount of RNA) of 20 s at 98°C, 30 s at 65°C, and 4 min at 72°C. Final elongation was performed for 5 min at 72°C. After PCR, the cDNA was purified twice with 0.6× SPRIselect beads (Beckman Coulter, CA). Next, 600 pg of preamplified cDNA were tagmented with Tn5 transposase (Lucigen) pre‐loaded with suitable adapters. Libraries were further amplified for 14 cycles using custom Nextera‐compatible primers with different indexes with KAPA HiFi HotStart ReadyMix. Libraries were loaded at 2.4 pM and sequenced on an Illumina Nextseq 500. The read configuration was 38 (read1), 8 (index1), 8 (index2), 38 (read2). The sequences of the oligonucleotides utilized are in Table S6.

### 4.10 RNA‐seq analysis

Smart‐seq reads were mapped using STAR 2.7.3a (Dobin et al., 2013) to the *Drosophila* genome assembly dm6. Unmapped and improperly paired reads were removed from bam files. Reads per gene annotated in the FlyBase release 2019_05 were computed using an in‐house R script based on the GenomicRanges (Lawrence et al., 2013) function summarizeOverlaps, which counts the number of reads overlapping with the exons of each gene in the default “union” mode. Normalized count matrices and differential expression analysis were performed using DESeq2 (Love et al., 2014). PCA plots were made using the plotPCA function in DESeq2, with variance stabilized counts as the input. Differentially expressed genes were considered to be any gene with a *p*‐adjusted values of <0.05.

### 4.11 Heatmaps

Heatmaps were constructed using the pheatmap R package. Normalized counts for genes with differential expression (*p*‐adjusted <0.05) between Day 5 and Day 70 of either of the ensheathing glia driver lines were visualized, with values scaled by row. The comparison of mouse microglia with low or high LD content (Marschallinger et al., 2020) was done by first computing homologous genes. The top homolog for each fly gene and each mouse gene was determined using BLASTp, retaining hits with an *e*‐value <0.01. Only gene pairs with 1–1 mapping (reciprocal top hits) were considered in the final analysis. Normalized expression levels were divided by the overall mean for the species (all fly expression divided by the mean fly expression, and all mouse expression divided by mean mouse expression) for ease of visualizing the two species on the same heatmap.

## AUTHOR CONTRIBUTIONS

L.S. conceived the study with R.B. and designed and performed most experiments with help from J.G. E.J.S. performed all bioinformatic analyses. M.S. and L.J. performed imaging experiments. C.N.B. and F.C. performed histology in N.B.'s laboratory. S.L.B. obtained funding and supervised L.J.'s work. L.S., EJ.S., and R.B. wrote the manuscript with help from all authors.

## CONFLICT OF INTEREST STATEMENT

None declared.

## Supporting information


Figure S1–S6

Table S1–S6

## Data Availability

Next generation sequencing data generated for this study have been deposited in the NCBI GEO with accession number GSE179604. All full resolution imaging files are available at https://figshare.com/s/cd2779665a9ff9662f2f.
