# Supplementary material for: Ensheathing glia promote increased lifespan and healthy brain aging
Source: Aging Cell. 2023 Feb 24;22(5):e13803. doi: 10.1111/acel.13803 (PMC10186613; doi:10.1111/acel.13803)
Supplement: Supplementary file 1 — Figure S1–S6 [file ACEL-22-e13803-s001.pdf]

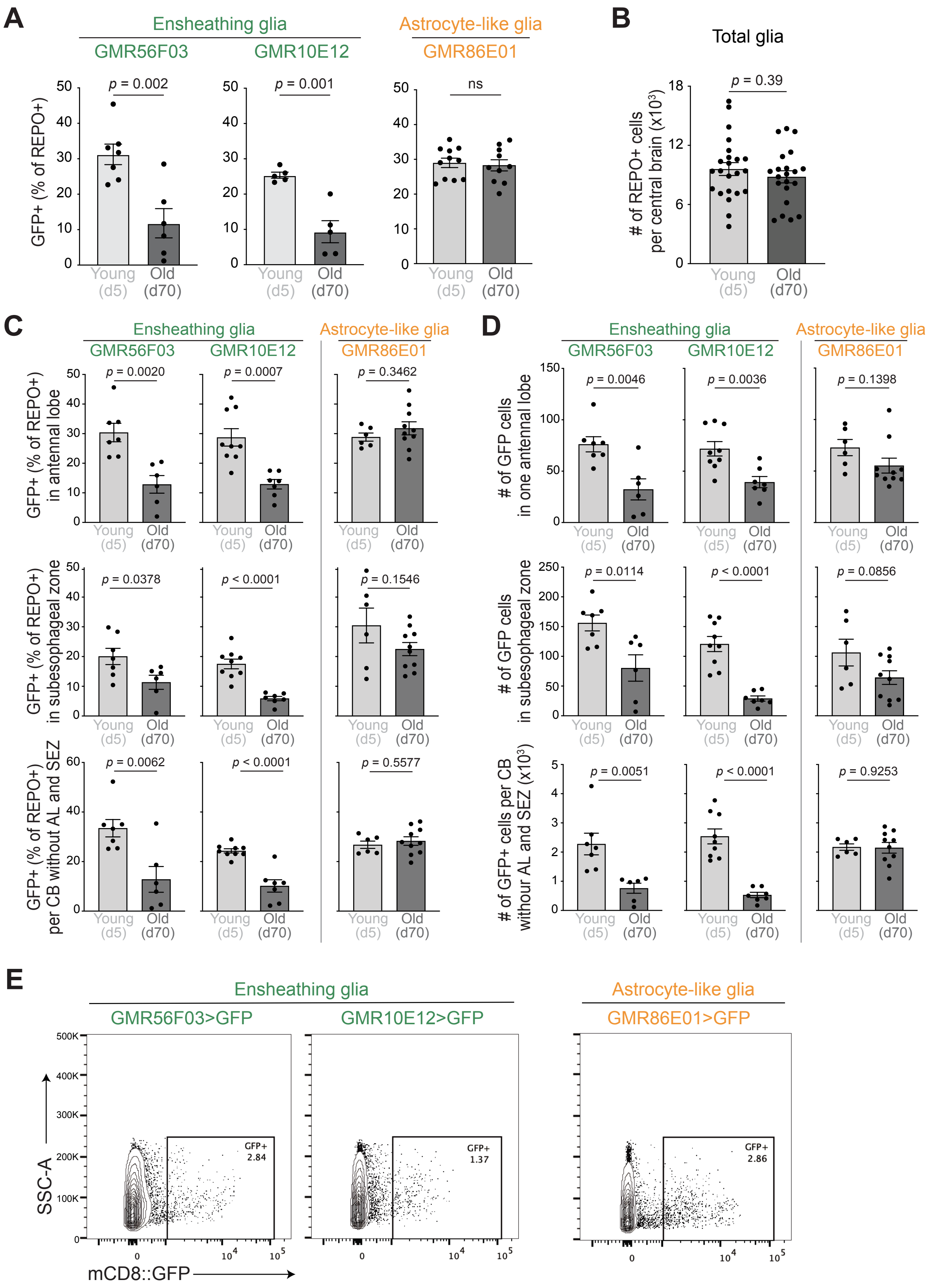

Figure S1. Additional quantifications of glia subsets

(A–D) Numbers of various glia subsets in young (day 5, light gray) and old (day 70, dark gray) fly brains as determined by microscopy. Cells were quantified as total GFP+ numbers per central brain (B, D) or % of GFP+ cells over REPO+ cells (A, C). Regions analyzed were central brain (A–B), antennal lobes (C–D top), subesophageal zone (C–D, middle), and central brain excluding antennal lobe and subesophageal zone (C–D, bottom). Indicated glia subset drivers (A, C–D) or total REPO+ glial cells (B) were quantified. Individual dots represent independent biological replicates (different brains). Bars represent mean ± SEM. p values are from Student's t tests.

(E) Representative flow cytometry data for the quantifications shown in Fig. 1D and the RNA-seq experiments shown in Fig. 2. Brains from young (day 5) flies are shown as examples.

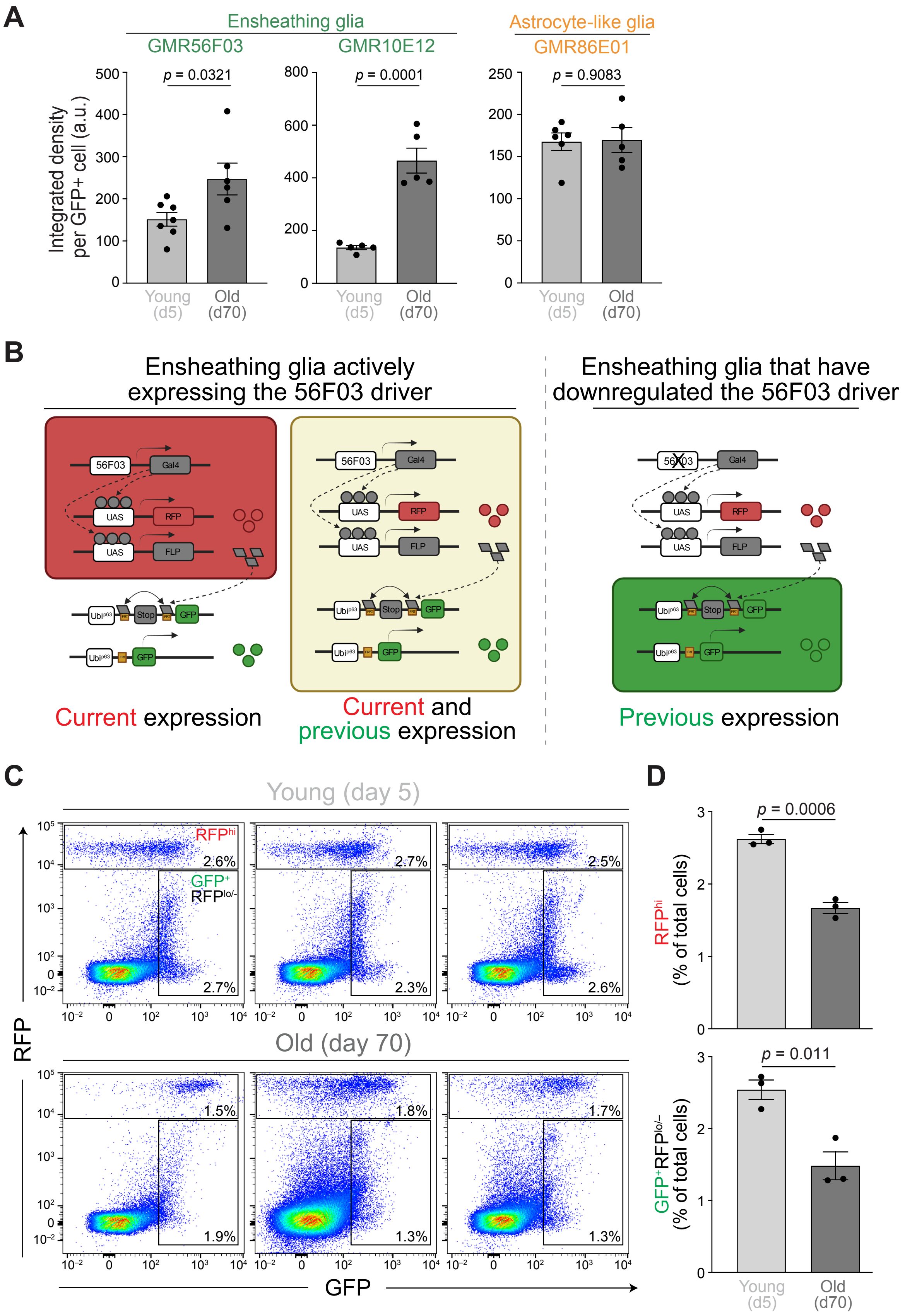

Figure S2. Driver expression analysis and lineage tracing

(A) Bar plots show the average integrated density of GFP expression per cell. Each dot represents the mean level per GFP+ cell in one individual brain. Bars represent mean ± SEM. p values are from Student's t tests.

(B) Scheme of the G-TRACE experiment indicating the identity of the different potential populations detected. Ensheathing glia cells actively expressing the 56F03 driver should be RFP+ and GFP- or GFP+ (left and middle), depending on speed and efficiency of FLP-recombination, whereas cells previously expressing 56F03 that have downregulated the driver over time should be GFP+ and RFP-low or RFP- (right).

(C) Flow cytometry from brains of young (day 5) and old (day 70) GMR56F03>G-TRACE flies. Plots show the gating strategy for identifying RFP-high (currently expressing) and GFP+RFPlow/- (previously expressing) presumptive ensheathing glia cells. Three biological replicates per group are shown.

(D) Quantification of cells in the two populations shown in (C) as % of total live cells. P values are from Student's t tests.

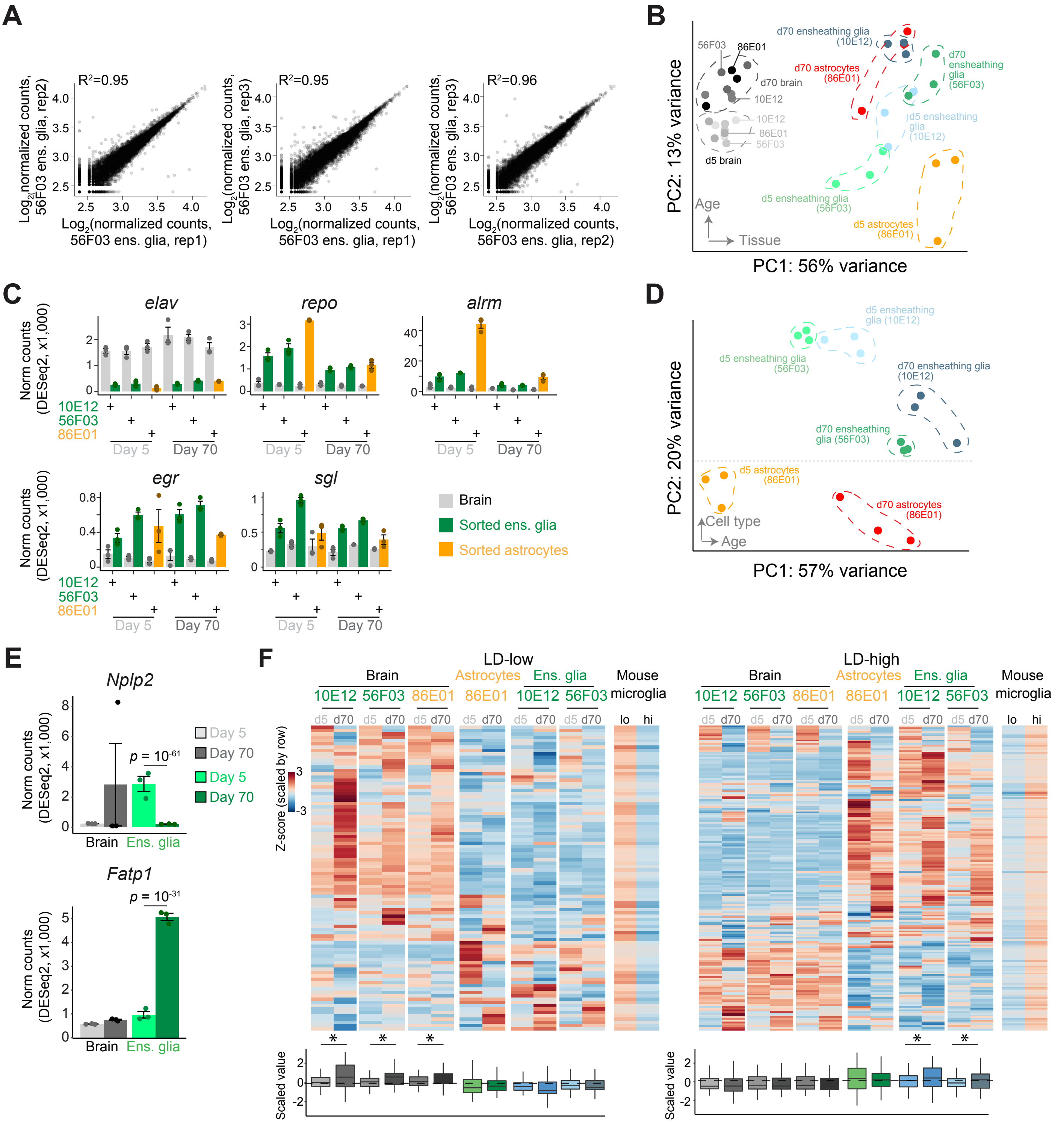

Figure S3. Validation of glia sorting for Smart-seq and evolutionary analyses

(A) Scatter plots comparing the normalized counts per gene from three biological replicates of one representative sample (sorted ensheathing glia from the GMR56F03 driver, day 5).

(B) PCA of genome-wide transcriptome profiles obtained by Smart-seq of whole brains or sorted glia using the indicated drivers. Each point represents one biological replicates. Different colors group represent different samples.

(C) Normalized Smart-seq counts for marker genes of neurons (elav), glia (repo), astrocyte-like glia (alrm), and ensheathing glia (egr and sgl). Individual biological replicase are represented by circles. Bars show mean ± SEM.

(D) PCA as in (B) only using ensheathing glia and astrocyte-like glia markers defined by single-cell RNA-seq (Davie et al., 2018) and only showing data points for sorted glia. Each circle represents one biological replicates. Different colors group represent different samples.

(E) Expression (normalized counts) of *Nplp2* and *Fatp2* in the whole brain and sorted ensheathing glia in young and old flies. Bars show the mean ± SEM.

(F) Expression levels in total brain and different glia populations of genes homologous to those downregulated (left) or upregulated (right) in mouse microglia that accumulates lipid droplets in old age. The heatmap colors represent Z-score of normalized counts divided by the species-wide mean for each gene, scaled by row.

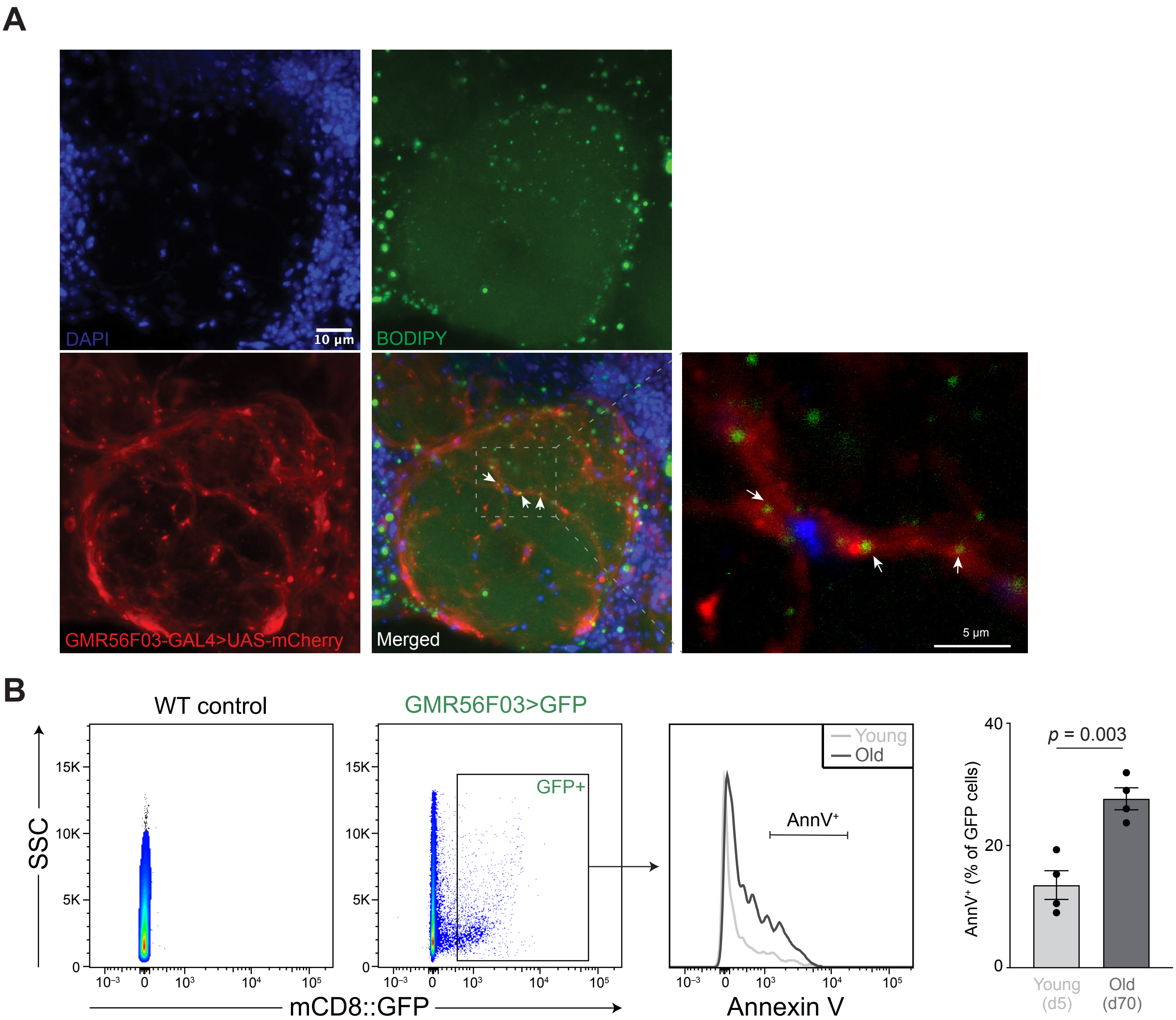

Figure S4. LDs and apoptosis in young and old ensheathing glia

(A) Microscopy images showing co-localization (white arrows) of ensheathing glia (GMR56F03>mCD8::mCherry, red) and the lipid stain BODIPY (green) in a Drosophila antennal lobe. The bottom right panel shows a higher magnification of the region in the dashed gray box.

(B) Quantification of apoptosis in ensheathing glia by flow cytometry. Left: gating strategy showing GFP expression in a WT control and GMR56F03>GFP flies (5-day old). Middle: histogram showing annexin V staining in ensheathing glia in a representative young (light gray) and old (dark gray) brain. Right: bar plots showing the % of ensheathing glia (GFP+) cells that were positive for annexin V. Each circle is a biological replicate. Bars show the mean ± SEM. P value is from a Student's t test.

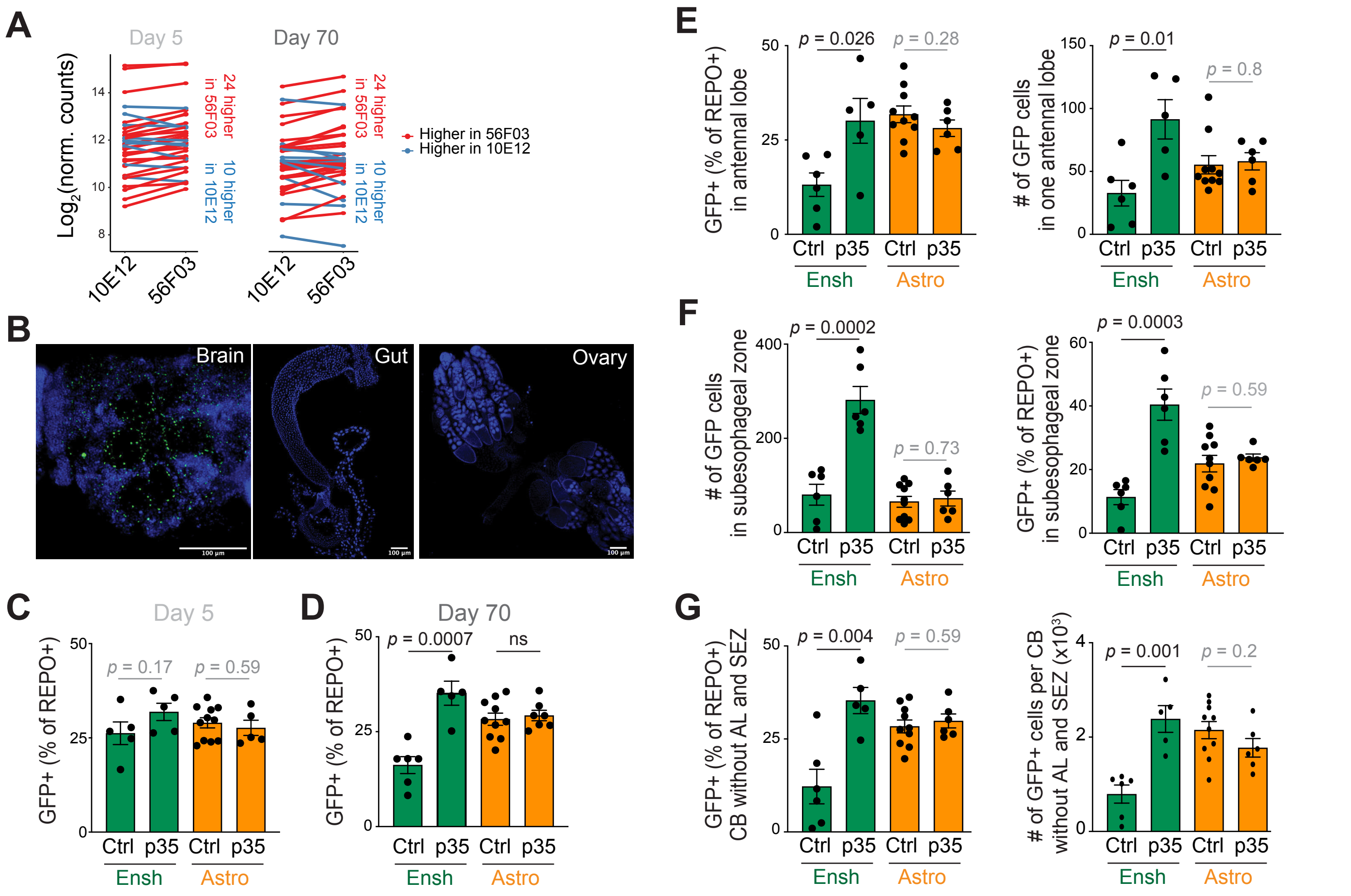

H

Genotype	Sex	*N*	Med life	Max life
+; UAS35	F	100	64	85
GMR56F03-GAL4; +	F	121	62	85
Iso31 (WT)	F	100	62	85
GMR56F03-GAL4>UAS-GFP	F	136	57	85
GMR56F03-GAL4>UAS-p35	F	115	70	95
+; UAS-p35	M	125	64	90
GMR56F03-GAL4; +	M	109	64	85
Iso31 (WT)	M	128	63	83
GMR56F03-GAL4>UAS-GFP	M	121	60	85
GMR56F03-GAL4>UAS-p35	M	171	70	90

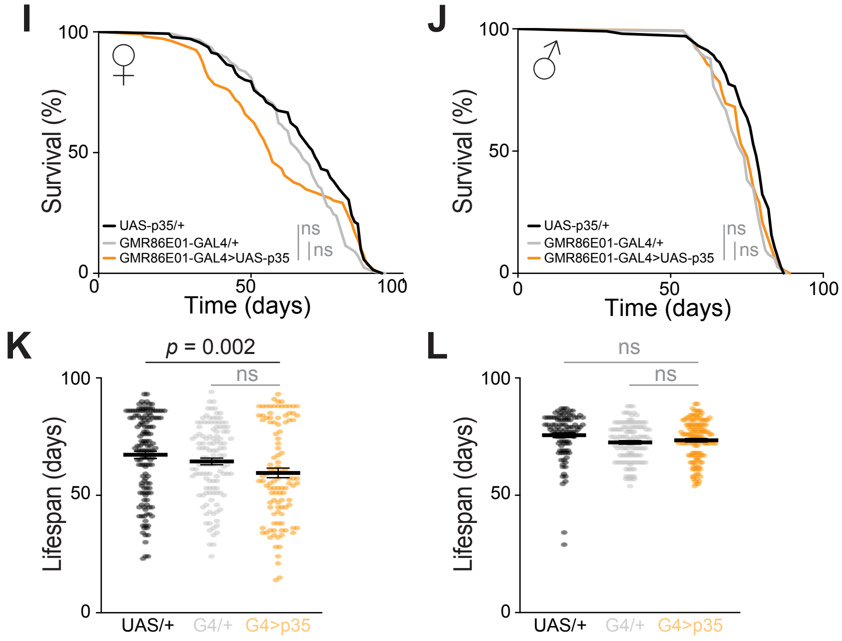

Figure S5. Additional analyses on lifespan

(A) Comparison of mRNA levels (DESeq2 normalized counts) for 34 ensheathing glia marker genes in ensheathing glia cells sorted by GFP expression under the GMR10E12 or GMR56F03 driver.

(B) Microscopy images for the expression (or lack thereof) of the GMR56F03 driver in adult brain gut and ovary. Scale bars correspond to 100 µm.

(C–G) Quantification of ensheathing glia cells (left, green bars) or astrocytes (right, orange bars) in 5-days-old (C) and 70-days-old (D–G) brains, with or without expression of p35. Numbers of ensheathing glia cells are expressed as % of total REPO+ glia or total number per sample, as indicated. Points represent biological replicates (individual brains). Bars represent mean ± SEM. p values are from Student's t tests.

(H) Summary table with all genotypes and values from the lifespan analyses in Fig. 3C–F. N, number of flies analyzed. "Max life" indicates the lifespan of the longest living individual in each group.

(I–J) Lifespan of female (I) or male (J) flies expressing p35 in astrocytes and relevant genetic controls. The 86E01>p35 curve was compared against the controls with logrank Mantel-Cox tests adjusted for multiple comparisons with the Bonferroni method. No significant ($p < 0.05$) differences were found.

(K–L) Lifespan distribution of individual females (K) and male (L) flies expressing p35 in astrocytes compared to control flies. Black bars represent the mean ± SEM. p values are from one-way ANOVA and Bonferroni multiple comparisons test.

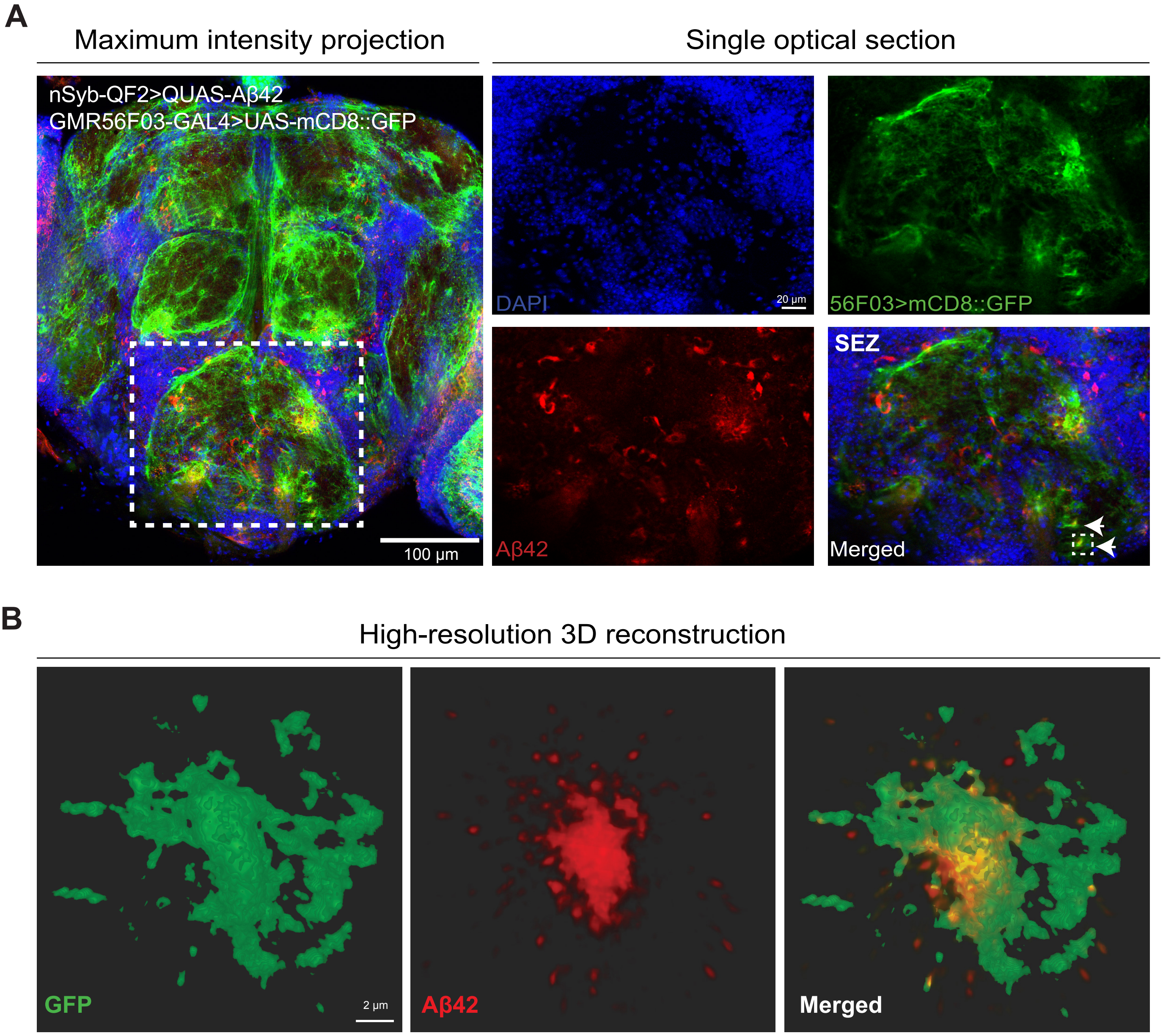

Figure S6. Double-labeling immunofluorescence analyses of co-staining of Aβ42 deposit and ensheathing glia

(A) Left: Z-stack projection of confocal microscopy images showing co-localization of Aβ42 signal (red) with a membrane marker expressed in ensheathing glia (56F03>mCD8::GFP). Dashed line indicates the subesophageal zone (SEZ). Right: a single optical section at a higher magnification and with separate channels of the SEZ. Arrows point to the ensheathing glia-co staining with Aβ42. Scale bar corresponds to 100 µm (left) and 20 µm (right). Dashed square with arrow indicates the area magnified in (B).

(B) 3D reconstruction in high magnification of the dashed square in the SEZ from (A) showing Aβ42 signal inside an ensheathing glia cell as determined by mCD8::GFP expression. Scale bar corresponds to 2 µm.
